# Effects of interval between age at first pregnancy and age at diagnosis on breast cancer survival according to menopausal status: a register-based study in Korea

**DOI:** 10.1186/1472-6874-14-113

**Published:** 2014-09-18

**Authors:** JungSun Lee, Minkyung Oh, The Korean Breast Cancer Society

**Affiliations:** Department of Surgery, College of Medicine, University of Inje, Haeundae paik Hospital, Busan, #1435, Jwa-dong, Haeundae-gu, Gimhae Zip-code: 612-030 Korea; Department of Pharmacology, Clinical Trial Center, Inje University Busan Paik Hospital, Bokji-ro 75, Busanjin-gu, Busan Zip-code: 614-735 Korea

**Keywords:** Reproduction, Breast cancer survival, Menopause

## Abstract

**Background:**

The influence of parity and time interval between age at first pregnancy (AFP) and age at diagnosis on breast cancer survival is not established in the same way as their influence on breast cancer risk. We aimed to investigate the association of time interval or parity with prognosis in pre- and postmenopausal women in Korea.

**Methods:**

We conducted a retrospective study of 29,167 women with breast cancer through the Korean Breast Cancer Registry from 1993–2009. Information on reproductive factors, including breastfeeding, AFP, and parity were collected from a routine questionnaire. Conditional logistic regression was used to estimate the associations between menopausal status and overall mortality (OM) and breast-cancer-specific mortality (BCSM), adjusting for treatment and stage.

**Results:**

High parity (≥5) increased the hazard ratios (HR) of BCSM (HR = 1.33, 95% confidence interval (CI): 0.83–2.11, p < 0.001) and OM (HR = 1.20, 95% CI: 0.85–1.68.73, p < 0.001) in premenopausal and postmenopausal women (BCSM, HR: 1.62, 95% CI: 0.93–2.82, p < 0.001; OM, HR = 1.58, 95% CI: 1.14–2.21, p <0.001). A longer time interval between age at breast cancer diagnosis and AFP reduced the HRs of BCSM (HR = 0.97, 95% CI: 0.96–0.98, p = 0.001) and OM (HR = 0.98, 95% CI: 0.97–0.98, p < 0.001) in premenopausal women, but had an adverse effect on the HR of OM (HR = 1.03, 95% CI: 1.02–1.03, p < 0.001) in postmenopausal women.

**Conclusions:**

High parity (≥5) was associated with poor breast cancer prognosis in both pre- and postmenopausal women. The time intervals between reproductive events had different effects on breast cancer outcomes depending on menopausal status.

**Electronic supplementary material:**

The online version of this article (doi:10.1186/1472-6874-14-113) contains supplementary material, which is available to authorized users.

## Background

An early age at first pregnancy has been shown to be protective in terms of lifetime risk of breast cancer. This protective effect may be explained by the differentiation of breast tissues induced by a full-term pregnancy, resulting in lower susceptibility to carcinogenic influences [1]. However, events considered to be protective in terms of breast cancer risk, such as early age at first pregnancy and increased parity, may have an adverse effect on breast cancer progression in women who are diagnosed with the disease. It is possible that the timing of reproductive events in relation to the initiation and promotion of breast cancers may be critical in determining their effects in women who develop breast cancer during their principal reproductive years [2].

Epidemiological studies have suggested that the endogenous host environment, including reproductive history, body mass index, and BRCA germline mutation, may correlate with breast cancer features and prognosis [3–11]. However, retrospective case–control studies of the relationship between breast cancer survival and host-related reproductive factors have tended to report inconsistent results [12–14]. Reproductive factors may induce permanent changes in the mammary gland epithelium or surrounding stromal tissue, and the most prominent effects may be related to the occurrence and timing of pregnancy [15, 16].

It is possible that the tissue changes associated with pregnancy may make the breast more or less susceptible to carcinogenic factors [15], and the resulting effects may also depend on the underlying genetic susceptibility to breast cancer [17]. It is therefore possible that factors related to age at menarche, the timing of pregnancies, the prevalence of breastfeeding, and age at menopause may either initiate or inhibit specific types of breast cancers with different degrees of aggressiveness.

Information from the Korean Breast Cancer Society showed that reproductive factors, including early menarche, late menopause, late first birth and no breastfeeding increased steadily in Korean women with breast cancer from 1996 to 2004 [18]. Reproductive events associated with tumor initiation or progression mostly occurred in the premenopausal period. Furthermore, the proportion of premenopausal breast cancer in Korea was higher than in Western countries [12, 18]. We previously reported the relationship between reproductive breast risk factors and breast cancer survival by breast cancer subtypes [19]. High parity (≥4) and early age at first pregnancy (<20 years) were correlated with worse outcomes in patients with luminal breast cancer, but not with other subtyped breast cancers. The time intervals between reproductive events, such as age at menarche, AFP, and age at diagnosis, may also differ according to menopausal status, which may also affect the impact of these factors on clinical outcome. The present study examined the impact of host-related reproductive factors on breast cancer prognosis according to pre- or postmenopausal status.

## Methods

### Study population

Patients diagnosed with breast cancer who were registered in the Korean Breast Cancer Society Registry between January 1993 and December 2009 were studied retrospectively. 71.7% of Korean breast cancer patients were registered in the Korean Breast Cancer Society Registry. A total of 29,167 patients were registered by 102 general hospitals, including 41 university hospitals and 61 surgical training hospitals. The database provided information about sex, age, type of operation, stage according to the 6th American Joint Committee on Cancer (AJCC) classification, histological findings, and presence of biological markers, adjuvant therapy, survival, and the cause of death, which was obtained from the Ministry of Health and Welfare, Republic of Korea. The Korean Breast Cancer Society Registry has been described in detail elsewhere [12]. The following exclusion criteria were used: (a) male, (b) women who gave birth after breast cancer diagnosis, and (c) no available reproductive data. Information on patient age, demographics, reproductive variables (age at menarche, pregnancy and childbirth history, breastfeeding, AFP, use of oral contraceptives, and use of hormone-replacement therapy) were collected from personal interviews conducted with each patient at the time of diagnosis. Participants were considered to be postmenopausal if they reported that their menstrual cycles had stopped for at least 12 months prior to their breast cancer diagnosis. Body mass index was calculated based on body weight at diagnosis and the reported maximum body height. Use of hormone replacement therapies was defined as never, former use, or current use. The tumor characteristics at the time of diagnosis (tumor size, histology, stage, and estrogen receptor (ER), progesterone receptor (PR), and HER2 expression) were determined on the basis of pathology reports in 28,989 of the patients who were registered in the cancer registry database. The tumors were staged using the TNM staging system, which follows the AJCC criteria and involves the assignment of appropriate letters or numbers to the following three fields: T (primary tumor), N (nodal involvement), and M (distant metastasis) [20]. This classification was recorded for both clinical and pathological staging. ER and PR expression were recorded as either negative or positive, while a HER2 status of 0, 1+ or 2+ was considered negative and 3+ was considered positive. This study was approved by the ethics committee at University of Inje.

### Statistical analyses

Patient groups were compared using χ^2^ tests for discrete data. The results of all tests were considered significant at p values of <0.05. Multivariate Cox proportional hazard models were used to determine the effects of reproductive factors on breast-cancer-specific mortality, (BCSM; death due to breast cancer) and overall mortality (OM;death from any cause). All analyses were adjusted for potential prognostic factors and stratified for menopausal status. Hazard ratios (HR) and 95% confidence intervals (CI) were calculated for the following study factors: AFP (<20 years, 20–24, 25–29 years, ≥30 years); number of children before diagnosis (0, 1, 2, 3, 4, ≥5); and breastfeeding history in parous women (never, ever). Trends in time intervals between age at diagnosis and AFP, or between AFP and menarche were determined by linear regression for continuous data. All analyses were performed using SAS version 8.2 (SAS institute Inc., Cary, NC, USA). In this manuscript we confirm that our research have adhered to the STROBE guidelines (Additional file 1).

## Results

### Reproductive factors and tumor characteristics

Mean age of total patients was 48.3 (±10.5) years old, that of premenopausal patients was 44.8 (±9.1) years old, and that of postmenopausal patients was 57.7 (±8.0) years old.

T1, T2 staged breast cancer was more frequent in postmenopausal than in premenopausal breast cancer patients (84.9% versus 82.3%, p < 0.001). Lymph node metastasis (39.8% versus 38.0%, p < 0.001) and ER positivity (60.7% versus 57.1%, p < 0.001) were more frequent in premenopausal breast cancer patients, while Her-2/neu positivity was more common in postmenopausal than in premenopausal breast cancer patients (24.5% versus 23.2%, p = 0.026). Early AFP (≤24 years) (17.1% versus 44.4%, p < 0.001), and high parity (≥5) (1.3% versus 9.7%, p < 0.001) were more frequent in postmenopausal breast cancer patients (Table 1).

**Table 1:** **Baseline characteristics at diagnosis among all breast cancer patients stratified by menopausal status**

	All women		Premenopausal		Postmenopausal		p-value
	n	%	n	%	n	%	
T stage							<0.001
T1,2	24051	82.9	17378	82.3	6673	84.9	
T3,4	2235	17.1	1682	17.7	553	15.1	
LN involvement							<0.001
(-)	17591	60.7	12714	60.2	4877	62.0	
(+)	11398	39.3	8412	39.8	2986	38.0	
ER							<0.001
(-)	10701	40.3	7527	39.3	3174	42.9	
(+)	15829	59.7	11608	60.7	4221	57.1	
HER2							0.026
(-)	18217	76.5	13263	76.9	4954	75.5	
(+)	5605	23.5	3996	23.2	1609	24.5	
Age at first pregnancy (years)							<.0001
Nulliparous	4943	19.08	4441	23.76	520	6.95	
<20	520	2.01	202	1.08	318	4.41	
20-24	6653	25.68	3756	20.10	2897	40.13	
25-29	10923	42.16	8114	43.41	2809	38.91	
≥30	2871	11.08	2178	11.65	693	9.60	
Parity							<.0001
Nulliparous	4943	17.05	4441	21.02	502	6.38	
1	3874	13.36	3095	14.65	779	9.91	
2	13269	45.77	10458	49.50	2811	35.75	
3	4345	14.99	2363	11.19	1982	25.21	
4	1523	5.25	496	2.35	1027	13.06	
≥5	1035	3.57	273	1.29	762	9.69	

Association between reproductive factors and breast cancer survival stratified by menopausal status.

### AFP

Compared with nulliparity, any AFP decreased the HRs of BCSM and OM in premenopausal breast cancer patients. However, early AFP (≤20 years) and late AFP (≥30 years) increased the HRs of BCSM (HR = 1.49, 95% CI: 0.76–2.89 and HR = 1.50, 95% CI: 0.85–2.67, respectively) and OM (HR = 1.40, 95% CI: 0.94–2.09 and HR = 1.18, 95% CI: 0.83–1.68, respectively) (p <0.001) in postmenopausal breast cancer patients (Table 2). The median (±standard deviation) follow-up duration was 13.4 (±1.9) years in premenopausal women, and 11.7 (±4.2) years in postmenopausal women.

**Table 2:** **Risks of breast-cancer-specific and overall survival according to age at first pregnancy stratified by menopausal status**

	Premenopausal			Postmenopausal		
	Case	Events	HR (95% CI)	Case	Events	HR (95% CI)
AFP	Breast cancer specific survival					
Nulliparous	4441	269	Ref	502	17	Ref
<20	202	5	0.436 (0.180-1.057)	318	18	1.492 (0.769-2.896)
20-24	3756	100	0.429 (0.341-0.540)	2897	116	1.090 (0.655-1.813)
25-29	8114	193	0.376 (0.312-0.452)	2809	88	0.834 (0.496-1.403)
≥30	2178	51	0.372 (0.275-0.501)	693	38	1.508 (0.851-2.673)
p-value			<.0001			0.0172
AFP	Overall survival					
Nulliparous	4441	561	Ref	502	47	Ref
<20	202	16	0.672 (0.409-1.105)	318	49	1.404 (0.941-2.095)
20-24	3756	274	0.551 (0.477-0.637)	2897	332	1.076 (0.793-1.460)
25-29	8114	513	0.468 (0.415-0.528)	2809	231	0.748 (0.546-1.023)
≥30	2178	159	0.547 (0.459-0.653)	693	87	1.183 (0.830-1.687)
p-value			<.0001			<.0001

### Parity

Compared with nulliparity, high parity (≥5) significantly increased the HRs of BCSM (HR = 1.32, 95% CI: 0. 83–2.11) and OM (HR = 1.20, 95% CI: 0.85–1.68) in premenopausal women, and also in postmenopausal women (p <0.0001) (Table 3). The median follow up duration was 13.4 (±1.9) years in premenopausal women and 11.7 (±4.2) years in postmenopausal women.

**Table 3:** **Risks of breast-cancer-specific and overall survival according to parity stratified by menopausal status**

	Premenopausal			Postmenopausal		
	Case	Events	HR (95% CI)	Case	Events	HR (95% CI)
Parity	Breast cancer specific survival					
Nulliparous	4441	269	Ref	502	17	Ref
1	3095	87	0.463 (0.363-0.590)	779	36	1.338 (0.752-2.383)
2	10458	257	0.386 (0.326-0.458)	2811	100	0.986 (0.590-1.650)
3	2363	84	0.574 (0.449-0.733)	1982	57	0.750 (0.436-1.289)
4	496	27	0.905 (0.609-1.345)	1027	42	1.079 (0.614-1.896)
≥5	273	19	1.329 (0.835-2.117)	762	48	1.622 (0.933-2.821)
p-value			<.0001			0.0029
Parity	Overall survival					
Nulliparous	4441	561	Ref	502	47	Ref
1	3095	252	0.641 (0.552-0.743)	779	81	1.056 (0.737-1.513)
2	10458	699	0.497 (0.444-0.555)	2811	231	0.806 (0.589-1.103)
3	2363	178	0.570 (0.482-0.675)	1982	174	0.789 (0.572-1.090)
4	496	57	0.880 (0.670-1.155)	1027	123	1.065 (0.761-1.491)
≥5	273	36	1.204 (0.859-1.687)	762	139	1.587 (1.140-2.210)
p-value			<.0001			<.0001

### Breastfeeding

Breastfeeding had no significant effect on the HR of either BCSM (HR = 1.08, 95% CI: 0.89–1.31, p = 0.42) or OM (HR = 0.91, 95% CI: 0.811.03, p = 0.14) in premenopausal or postmenopausal breast cancer patients (BCSM, HR = 0.80, 95% CI: 0.58–1.10, p = 0.17; OM, HR = 0.98, 95% CI: 0.80–1.20, p = 0.86) (Table 4).

**Table 4:** **Risks of breast-cancer-specific and overall survival according to breastfeeding history stratified by menopausal status**

	Premenopausal			Postmenopausal		
	Case	Events	HR (95% CI)	Case	Events	HR (95% CI)
Breast feeding	Breast cancer specific survival					
Yes	10794	297	1.083 (0.890-1.319)	6176	218	0.803 (0.586-1.101)
No	5820	149	Ref	1122	47	Ref
p-value			0.4267			0.1726
Breast feeding	Overall survival					
Yes	10794	751	0.916 (0.814-1.030)	6176	619	0.982 (0.800-1.206)
No	5820	445	Ref	1122	107	Ref
p-value			0.1412			0.8612

### Time interval between reproductive events

A longer time interval between AFP and menarche significantly decreased the HR of BCSM (HR = 0.96, 95% CI: 0.93–0.99, p < 0.009) but had no effect on the HR of OM (HR = 0. 99, 95% CI: 0.97–1.00, p = 024) in premenopausal breast cancer patients (Table 5). A longer time interval between AFP and age at diagnosis reduced the HRs of BCSM (HR = 0.97, 95% CI: 0.96–0.98, p < 0.001) and OM (HR = 0.98, 95% CI: 0.97–0.98, p < 0.001) in premenopausal breast cancer patients, but had no significant effect on BCSM, but a longer time interval between AFP and age at diagnosis increased the HR of OM in postmenopausal breast cancer patients (HR = 1.03, 95% CI: 1.02–1.03, p < 0.001) (Table 6).

**Table 5:** **Risks of breast-cancer-specific and overall survival according to time interval from AFB to menarche**

	Premenopausal			Postmenopausal		
	Case	Events	HR (95% CI)	Case	Events	HR (95% CI)
AFB-age of menarche	Breast cancer specific survival					
	14057	339	0.965 (0.939-0.991)	6579	251	0.983 (0.955-1.013)
p-value			0.0095			0.2621
AFB-age of menarche	Overall survival					
	14057	938	0.990 (0.975-1.007)	6579	678	0.961 (0.943-0.979)
p-value			0.2455			<.0001

**Table 6:** **Risks of breast-cancer-specific and overall survival according to time interval from AFB to age at diagnosis**

	Premenopausal			Postmenopausal		
	Case	Events	HR (95% CI)	Case	Events	HR (95% CI)
Age at diagnosis-AFB	Breast cancer specific survival					
	14387	352	0.975 (0.962-0.989)	6761	261	1.005 (0.992-1.018)
p-value			0.0003			0.4578
Age at diagnosis-AFB	Overall survival					
	14387	969	0.981 (0.973-0.989)	6761	701	1.031 (1.023-1.038)
p-value			<.0001			<.0001

## Discussion

A relatively young AFP is primarily associated with a reduced risk of lower grade, ER-positive breast cancer [3, 20], which is generally associated with better survival than high-grade, ER-negative tumors [21]. This suggests that women with a young AFP who develop breast cancer may be more likely to have ER-negative, high-grade tumors, which could explain the association between early first pregnancy and poor prognosis reported in some studies. Early age at diagnosis has also been associated with a poor prognosis in some studies [22–24]. However, the biologic processes underlying these findings are not well understood, and probably involve a combination of inheritance [25], tumor gene expression in young women [21], and the hormonal milieu in premenopausal compared with postmenopausal women. In contrast, a late age at first pregnancy has been associated with higher risk of more aggressive ER-positive breast tumors in some studies [26, 27]. Therefore, both early and late AFP may be associated with poor survival, though the underlying biological mechanisms may differ.

The results of this study suggest that parity, irrespective of AFP, was associated with a more favorable prognosis in terms of BCSM and OM compared with nulliparity in premenopausal women, though both early (<22 years) and late AFP may be associated with poor survival in postmenopausal women. Some studies reported no associations [27, 28], while others found that poorer survival was associated with an early AFP [23, 29]. Alsaker et al. [30] reported that late AFP may be associated with a poorer prognosis among women with postmenopausal breast cancer.

Pregnancy-related factors may result in long-term changes in the hormonal milieu [31]. However, although high parity clearly protects against breast cancer [32], its protective effect may be limited to ER-positive tumors [3], and it has been suggested that high parity may conversely increase the risk of triple-negative breast cancer [30], which is known to have a poor prognosis [9] and to be more common in younger women [33]. A similar but smaller study also found that high parity may have a protective effect against small and low-grade tumors [25, 26], with the consequence that women with high parity may tend to have relatively advanced and aggressive disease. In this study, high parity (≥5) increased the HRs of BCSM and OM in premenopausal breast cancer patients, and this effect was attenuated beyond menopause. Compared with nulliparity, both single parity and high parity (≥5) were positively associated with high mortality from breast cancer in postmenopausal women, suggesting that the postmenopausal hormonal milieu may alter the protective effect of childbirth. In contrast to this, some studies demonstrated that nulliparous women had better survival than parous women, whereas the association was reversed in postmenopausal women [23, 34].

It is therefore unclear if the association between high parity and overall survival in the current study reflects a general decrease in female survival associated with high parity, as a result of deaths due to parity-related diseases such as cardiovascular disease, and postpartum related diseases, without specifically affecting the course of breast cancer. High parity might then be expected to have a greater effect on overall survival in postmenopausal women, in terms of mechanisms such as socioeconomic effects, and general health care [28, 29].

Several recent studies have shown that the first pregnancy induces a specific genomic signature in the breast epithelium [14, 35]; inflammation-associated genes were up-regulated in premenopausal parous human breast tissue, and Her-2/neu expression was changed relative to nulliparous human breast tissue from the same generation [35]. The genomic profile of breast cancer cases, irrespective of parity history, differed from those of parous or nulliparous cancer-free cases, according to hierarchical clustering [14]. These findings suggest that the breast cancer cells were already generated before pregnancy, and that pregnancy contributed to the prevention of mammary carcinogenesis. Parous subjects had significantly reduced expression of ERα (ERE, ESR1), PR, and ErbB2 (Her-2/neu), and 2-fold higher expression of ERβ (ESR2) compared with nulliparous subjects. These differences in the expression profiles of the hormone signaling genes ESR1, ESR2, PR, and ERBB2 indicate parity-mediated protective effects.

Lactation is believed to be associated with decreased circulating levels of estrogen and progesterone, and was hypothesized to be associated with earlier-stage tumors with less aggressive profiles. Lactation among parous women was not found to be associated with tumor stage, though parous women who had breastfed a child were more likely to have ER-positive and PR-positive tumors than were parous women who had never breastfed [3]. This effect would explain a small benefit associated with breastfeeding, given that the prevalence of ER-negative tumors may be lower among parous women who have breastfed compared with those who have never breastfed. Lactation was therefore associated with tumor markers indicative of better prognosis [36]. We were unable to assess these effects in relation to breastfeeding, but our results were in line with those of other studies that showed no consistent association between breast cancer and breastfeeding [37].

The time interval from childbirth until diagnosis has been related to the prognosis of breast cancer in other large registry-based studies [23, 29, 38]. Women diagnosed with breast cancer close to a pregnancy tend to be younger and have a more advanced stage of disease [39], higher histological grade [25], and are more likely to have ER-negative tumors [40]. Several studies have shown that breast cancer patients who gave birth shortly before diagnosis had poorer survival outcomes than nulliparous patients or those with a less recent childbirth [17, 41]. Even after adjusting for these well-known prognostic factors, including AJCC stage, hormone receptor and Her-2/neu statuses, and histological tumor grade, women who delivered within 2 years of breast cancer diagnosis had a 2-fold increased risk of death compared with nulliparous women [11].

During pregnancy and subsequent lactation, the breast tissue evolves from an immature to a fully-developed state, associated with rapid cell turnover during and after pregnancy. High parity might thus eventually enhance the initiation or progression of malignant tumor cells. Daling et al. [40] reported that women diagnosed with breast cancer less than 2 years after childbirth had a relatively high proportion of p53-positive, PR-negative, lymph node-positive and grade III tumors, which could result from the progression of highly malignant cells during and after pregnancy.

Several hypotheses have been proposed to explain the poor prognosis of young breast cancer patients who have recently given birth. The gestational hormones estrogen, progesterone, and insulin-like growth factor increase tumor cell proliferation [42], and the special hormonal environment of pregnancy may influence the biology of more aggressive tumor types. Asztalos et al. [35] investigated if pregnancy and involution were associated with gene expression changes in the normal breast, and whether such changes were transient or persistent. They found that 22% of the selected gene set related to immune/inflammation, extracellular matrix remodeling, angiogenesis, and hormone signaling was differentially regulated in nulliparous versus parous breast tissues.

Pregnancy is associated with persistent changes in gene expression in normal breast tissue, which may contribute to the protective and stimulatory effects of pregnancy on breast cancer risk. Pregnancy appears to have a lasting effect on gene expression, which is detectable for up to 10 years and possibly even longer. Recent evidence suggests that ERα and ErbB2 collaborate in mediating estrogen-induced mitogenesis, and down regulation of ErbB2 in parous women may also be linked to a protective effect [43]. Russo and coworkers [44] used cDNA microarray analysis of ethanol-fixed tissue from postmenopausal women and showed that many genes were differentially expressed between parous and nulliparous women, including a number of immune-related genes that were up-regulated in the parous group.

Although the number of patients in the current study was moderate, follow-up was almost complete, and the lower number of breast cancer deaths ensured a relatively high statistical power for detecting important differences. However, the strength of the detected associations was typically weak, and we cannot rule out the possibility that some of the findings of the study may be explained by chance. Another weakness of this study may be the lack of detailed clinical information, both at diagnosis and during follow-up.

This study analyzed the HRs after adjusting for reproductive factors and tumor characteristics, including hormone receptor and Her-2/neu status and T stage, but we cannot exclude the possibility of residual confounding. Nevertheless, any remaining confounder that could influence the results would need to be strongly associated with breast cancer survival, as well as the reproductive factors under study, and be simultaneously unrelated to the confounders included in the analyses.

## Conclusions

We found that early AFP (<20 years) and late AFP (≥30 years) were associated with poorer breast cancer survival in postmenopausal breast cancer patients, but any AFP showed better clinical outcomes than nulliparity in premenopausal breast cancer patients. High parity (≥5) was associated with poorer clinical outcomes in pre- and postmenopausal women with breast cancer. However, we found no clear relation between breastfeeding and breast cancer survival in pre- or postmenopausal breast cancer patients.

Socioeconomic changes and pressures on young women in Korea mean that most younger women tend to have jobs and to delay getting married and having children, and therefore have a later AFP, and only breastfeed for a short period [19]. Further follow-up of existing prospective cohort studies is needed to confirm and clarify these relationships, especially for different tumor subtypes. In addition, more studies are needed to characterize the relationships between parity and various outcomes in breast cancer patients.

## Consent

Written informed consent was obtained from the patient for the publication of this report and any accompanying images.

## Electronic supplementary material

Additional file 1: **STROBE statement in reports of observational studies.** (DOC 84 KB)

## Abbreviations

AFP: Age at first pregnancy
BCSM: Breast-cancer-specific mortality
CI: Confidence intervals
ER: Estrogen receptor
HER2: Her-2/neu
HR: Hazard ratios
PR: Progesterone receptor
OM: Overall mortality.
